# Restoring FAS Expression via Lipid-Encapsulated FAS DNA Nanoparticle Delivery Is Sufficient to Suppress Colon Tumor Growth In Vivo

**DOI:** 10.3390/cancers14020361

**Published:** 2022-01-12

**Authors:** Alyssa D. Merting, Dakota B. Poschel, Chunwan Lu, John D. Klement, Dafeng Yang, Honglin Li, Huidong Shi, Eric Chapdelaine, Mitzi Montgomery, Michael T. Redman, Natasha M. Savage, Asha Nayak-Kapoor, Kebin Liu

**Affiliations:** 1 Department of Biochemistry and Molecular Biology, Medical College of Georgia, Augusta, GA 30912, USA; Amerting@augusta.edu (A.D.M.); Daposchel@augusta.edu (D.B.P.); Clu@augusta.edu (C.L.); Jklement@augusta.edu (J.D.K.); Dyang@augusta.edu (D.Y.); HLI@Augusta.edu (H.L.); 2 Georgia Cancer Center, Medical College of Georgia, Augusta, GA 30912, USA; Hshi@augusta.edu (H.S.); anayak@augusta.edu (A.N.-K.); 3 Charlie Norwood VA Medical Center, Augusta, GA 30904, USA; 4 Genprex Inc., Austin, TX 78746, USA; echapdelaine@genprex.com (E.C.); MMontgomery@genprex.com (M.M.); mredman@genprex.com (M.T.R.); 5 Department of Pathology, Medical College of Georgia, Augusta, GA 30912, USA; nsavage@augusta.edu

**Keywords:** FAS, colon cancer, metastasis, cytotoxic T lymphocyte, cationic lipid nanoparticle

## Abstract

**Simple Summary:**

A key feature of human colorectal tumor is loss of FAS expression. FAS is the death receptor for FASL of activated T cells. Loss of FAS expression therefore may promote tumor cell immune escape. We aimed at determining whether restoring FAS expression is sufficient to suppress colorectal tumor growth. Mouse and human FAS cDNA was synthesized and encapsulated into cationic lipid nanoparticle DOTAP-Cholesterol to formulate DOTAP-Chol-mFAS and DOTAP-Chol-hFAS, respectively. Restoring FAS expression in metastatic mouse colon-tumor cells enabled FASL-induced elimination of FAS^+^ tumor cells in vitro and suppressed colon-tumor growth and progression in tumor-bearing mice in vivo. Restoring FAS expression induced FAS receptor auto-oligomerization and tumor cell auto-apoptosis in metastatic human colon-tumor cells in vitro. DOTAP-Chol-hFAS therapy is also sufficient to suppress metastatic human colon tumor xenograft growth in athymic mice. Tumor-selective delivery of FAS DNA nanoparticle is potentially an effective therapy for human colorectal cancer.

**Abstract:**

A hallmark of human colorectal cancer is lost expression of FAS, the death receptor for FASL of cytotoxic T lymphocytes (CTLs). However, it is unknown whether restoring FAS expression alone is sufficient to suppress csolorectal-cancer development. The FAS promoter is hypermethylated and inversely correlated with FAS mRNA level in human colorectal carcinomas. Analysis of single-cell RNA-Seq datasets revealed that FAS is highly expressed in epithelial cells and immune cells but down-regulated in colon-tumor cells in human colorectal-cancer patients. Codon usage-optimized mouse and human FAS cDNA was designed, synthesized, and encapsulated into cationic lipid to formulate nanoparticle DOTAP-Chol-mFAS and DOTAP-Chol-hFAS, respectively. Overexpression of codon usage-optimized FAS in metastatic mouse colon-tumor cells enabled FASL-induced elimination of FAS^+^ tumor cells in vitro, suppressed colon tumor growth, and increased the survival of tumor-bearing mice in vivo. Overexpression of codon-optimized FAS-induced FAS receptor auto-oligomerization and tumor cell auto-apoptosis in metastatic human colon-tumor cells. DOTAP-Chol-hFAS therapy is also sufficient to suppress metastatic human colon tumor xenograft growth in athymic mice. DOTAP-Chol-mFAS therapy exhibited no significant liver toxicity. Our data determined that tumor-selective delivery of FAS DNA nanoparticles is sufficient for suppression of human colon tumor growth in vivo.

## 1. Introduction

The five-year survival rate for human colorectal cancer is 92%, but this rate drops to 7% if the cancer has metastasized to the liver. Metastasis accounts for more than 90% mortality in colorectal-cancer patients [1]. Advances in chemotherapeutic and biological agents, combined with liver resection, have increased the survival of patients with metastatic colorectal cancer to 50% [2]. However, only 20% of metastatic colorectal cancers are resectable, and the recurrence rate is ≈75% [3]. The mechanism underlying human colorectal-cancer progression and metastasis is still elusive, but down-regulation of the death receptor FAS is a hallmark of metastatic human colorectal cancer [4,5].

CD8^+^ cytotoxic T lymphocytes (CTLs) are the primary immune cells that kill tumor cells via the FAS-FASL [6,7] and perforin-granzyme [8] pathways. FAS is a death receptor expressed on the tumor cell surface, and its physiological ligand FASL is expressed on activated CTL and NK cell surfaces [7]. Although it has been reported that FAS also mediates non-apoptotic signaling pathways to promote tumor growth under certain cellular context [9], tumoral FAS expression plays a key role for CAR-T cell efficacy [10], and it alone predicts survival of chimeric antigen receptor (CAR)-T-treated patients in a large clinical trial [11]. Apoptotic extracellular vesicles with FASL induces myeloma FAS-mediated apoptosis to suppress tumor growth in vivo [12]. Compelling and emerging experimental data indicates that the FAS acts as a tumor suppressor in human cancer and is essential for CTL effector function in the suppression of tumor development [7,12,13,14,15,16,17,18,19,20,21,22,23,24,25,26]. In addition, the FAS-FASL pathway is essential for the cytotoxicity of CTLs, including CAR-T cells, in the elimination of tumors in both tumor antigen-specific and antigen-independent manners [11,14]. To avoid FAS-mediated apoptosis, human cancer cells often down-regulate FAS expression and may use it as a mechanism to evade cell death to survive and progress [27,28]. For example, it was reported that FAS is highly expressed in normal human colon epithelial cells but is down-regulated in human colorectal carcinoma, whereas complete loss of FAS expression is often observed in metastatic human colorectal tumor [4,5,29,30]. Therefore, FAS plays a key role in colorectal-cancer growth control carcinoma [4,23,29,30,31,32,33], and human colorectal cancer may use the silencing of FAS expression as a mechanism to progress and metastasize. However, it is unknown whether restoring FAS expression alone is sufficient to suppress metastatic colorectal tumors. 

FAS is highly expressed in human colon epithelial cells but down-regulated in human colorectal carcinoma [4,29,30]. It has been shown that tumor cells use an epigenetic mechanism, such as histone deacetylation, to silence FAS expression to evade host immune surveillance [20,34,35]. It was reported that the FAS promoter is hypermethylated in human colon-tumor-cell lines in vitro [36]. Furthermore, FAS is also repressed by its promoter H3K9me3 deposition in human colon-tumor cell lines, especially the metastatic human colon-tumor cells [37,38]. DNA hypermethylation, H3K9me3 deposition, and histone deacetylation may compensate for each other in repression of FAS expression [39], which may limit the efficacy of epigenetic agent monotherapy. Because increasing FAS expression and the FAS-mediated apoptosis pathway have been shown to increase the efficacy of CTL elimination of target tumor cells [11,13], we therefore aimed at testing the hypothesis that restoring FAS expression is sufficient to suppress metastatic colon-tumor growth. To this end, we designed and synthesized codon-optimized FAS cDNA and formulated cationic lipid-encapsulated FAS cDNA nanoparticles. We determined that codon optimization results in high FAS expression in colon-tumor cells, and tumor-selective delivery of FAS cDNA nanoparticles is sufficient to suppress metastatic colon-tumor growth in vivo.

## 2. Materials and Methods

### 2.1. Cell Lines

Murine colon carcinoma CT26 cell line (catalog #: CRL-2638), murine leukemia cell line EL4 (catalog #: TIB-39), human leukemia cell line Jurkat (catalog #: TIB-152), and human colon carcinoma cell lines SW480 (catalog #: CCL-228), SW620 (catalog #: CCL-227), and LS411N (catalog #: CRL-2159) were obtained from American Type Culture Collection (ATCC) (Manassas, VA, USA). Murine colon carcinoma cell line MC38 was provided by Dr. Jeffrey Schlom (National Cancer Institute, Bethesda, MD, USA). Cell lines were tested bi-monthly for mycoplasma and were mycoplasma-free at the time of experiments. All cells were maintained in an incubator at 37 °C with 5% CO_2_.

### 2.2. Mice

Mice were purchased from Jackson Laboratory (Bar Harbor, ME, USA). The use of mice in this study was approved by the Augusta University Institutional Animal Care and Use Committee (Protocol #: 20080162). All experiments were done with 3−5 mice per treatment group. Experiments were done in both male and female mice with ages ranging from 8 weeks to 12 weeks at start of all experiments.

### 2.3. Cationic Lipid Nanoparticle

Cationic lipid DOTAP-Cholesterol (1:1) is manufactured at T&T Scientific Corp (Knoxville, TN, USA). Codon-optimized FAS cDNAs was designed, synthesized, and cloned to NTC9385R nanoplasmid at Nature Technology Corp (Lincoln, NE, USA) [40]. Large-scale plasmid DNA preparation by fermentation was also carried out at Nature Technology Corp (Lincoln, NE, USA). Codon-usage-optimized mouse and human FAS cDNA and human cDNA is listed in Tables S1 and S5, respectively. To make DOTAP-Cholesterol-encapsulated plasmid DNA, DOTAP-cholesterol was diluted to 8–10 mM with 5% dextrose (Cat # 76313–652, VWR International). The plasmid DNA was also diluted to the desired concentration with 5% dextrose. The diluted DOTAP-Cholesterol and plasmid DNA were then mixed at 1:1 ratio and incubated at room temperature for 30 min. Each mouse received 100 mL encapsulated plasmid DNA via intravenous injection.

### 2.4. In Vivo Tumor Models

CT26 subcutaneous tumors were established by injecting CT26 cells (2 × 10^5^ cells/mouse) into the right flank of BALB/c mice. For survival study, CT26 cells (2 × 10^5^ cells/mouse) were injected into BALB/c mouse tail veins. SW620 subcutaneous xenografts were established by injecting SW620 cells (2.5 × 10^6^ cells/mouse) into the right flank of athymic mice. The cationic lipid nanoparticles were injected into mouse tail veins.

### 2.5. Single-Cell RNA Sequencing Dataset Analysis

Single-cell RNA sequencing datasets of human colorectal-cancer patients were extracted from the GEO database (GSE146771) [41]. Cells were annotated according to the dataset designation. Cells were subsetted by tissue of origin (normal colon, peripheral blood, and colorectal tumor), and the FAS expression level in the indicated cell types were determined using R package.

### 2.6. DNA-Methylation Analysis

Colorectal-cancer TCGA 450 K methylation array and RNA-seq data of FAS were downloaded from UCSC cancer genome browser (Xena). The normalized beta values and FPKM were used to generate the heatmap and correlation plots using ComplexHeatmap and ggpubr packages in R3.6.3. The promoter diagram was generated using karyoploteR. Statistical analyses were performed in R package.

### 2.7. Patient-Dataset Analysis

FAS mRNA datasets and survival datasets were extracted from the TCGA database. Survival analyses were performed using the R survival and survminer packages. 

### 2.8. Flow Cytometry

Samples were stained with fluorescent dye-conjugated anti-mouse FAS (clones: SA376h8, 554258, SA367H8), FASL (clone: MFL3), CD8 (clone: 53–6.7), and anti-human FAS antibodies (clone: DX2) (Biolegend, San Diego, CA, USA). All antibodies were used at a 1:100 dilution. Samples were acquired on an FACSCalibur with CellQuestPro or LSRFortessa with BD Diva 8.01 (BD Biosciences, San Diego, CA, USA). All flow cytometry data analysis was conducted with FlowJo v10.6.0 (BD Biosciences).

### 2.9. Cell-Death Analysis

Mega-FAS Ligand/APO010 was provided by Dr. Peter Buhl Jensen at Oncology Venture A/S, Denmark. Floating cells were collected from culture supernatant. Adherent cells were detached using trypsin and mixed with floating cells. The cells were suspended in annexin V-binding buffer (10 mM HEPES, pH 7.4, 140 mM NaCl, 2.5 mM CaCl_2_) and stained with APC-annexin V (Biolegend) and propidium iodide (PI). The stained cells were analyzed by flow cytometry.

### 2.10. Western Blotting

Tumor cells were lysed in total protein lysis buffer and separated in 4−20% SDS-polyacrylamide gels. The blots were probed with anti-human cleaved caspase 3 (catalog #: 9661s, 1:500 dilution), cleaved PARP (catalog #: 9544s) (Cell Signaling Tech, Danvers, MA, USA), FASL (catalog #: F37720, 1:1000 dilution) (BD Bioscience,), and b-actin (catalog #: A 5441, 1:5000 dilution) (Sigma-Aldrich, St Louis, MO, USA).

### 2.11. Transfection

CT26 and MC38 cells were transfected with the mFAS plasmid using the DOTAP-Chol (1:1); this was done by washing the cells in serum-free RPMI and then adding the DOTAP-Chol mFAS mixture to the cells and incubating them in serum-free RPMI for 3 h; then, the serum-free RPMI with complete RPMI were replaced and incubated for 24 h. Transfection rates were tested using flow cytometry. SW480, SW620, and LS411N cells were transfected with the hFAS plasmid using Lipofectamine 2000 Life Technologies, Danvers, MA, USA. Cells were tested for transfection efficacy using flow cytometry.

### 2.12. Statistical Analysis

Statistical analysis was conducted using Prism8 (Graphpad, San Diego, CA, USA), and *p*-values were calculated by a two-tailed Student’s t-test. Significance between survival groups was computed by a two-sided log-rank test using Survival package, R.

## 3. Results

### 3.1. FAS Is a Suppressor of Human Colorectal-Cancer Metastasis

**Figure 1:**
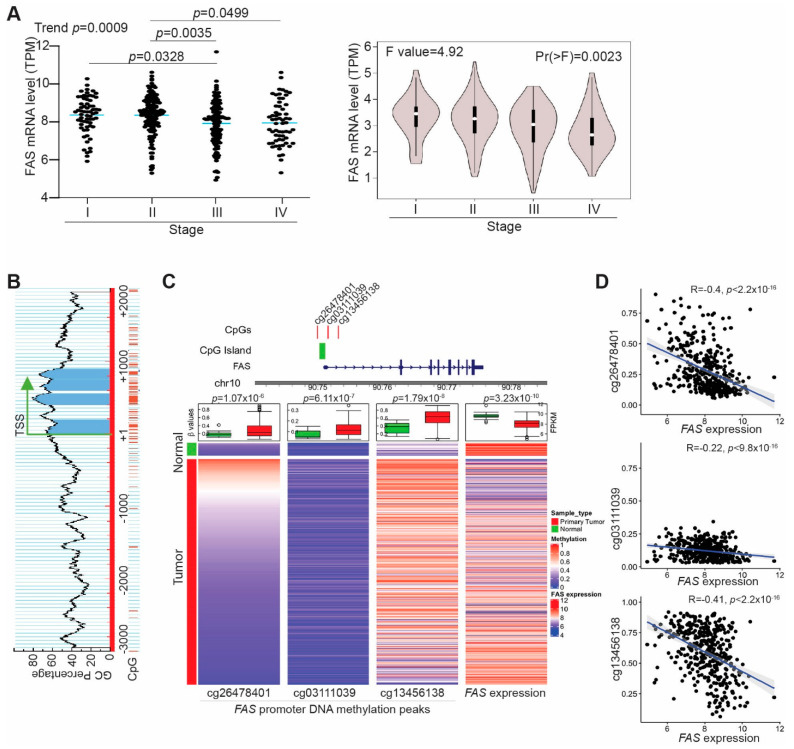
FAS **is silenced by its promoter DNA hypermethylation in human colorectal cancer.** (**A**) FAS mRNA data in human colorectal tumor were extracted from TCGA (left panel) and GEPIA (right panel) databases and grouped based on tumor stages. (**B**) The FAS promoter region showing CpG islands surrounding the FAS gene transcription start site. (**C**) Top panel (Left): The FAS promoter CpG island and three CpG sites significantly differentially methylated in TCGA colorectal cancer samples. Bottom panel (Left): Heatmaps of 450K DNA methylation array data of three CpG sites (cg26478401, cg03111039, cg13456138) and FAS expression (FPKM values) generated from TCGA human colorectal cancer datasets. The three columns on the left represent the beta values of the three CpG sites in primary colorectal cancer patient samples and normal colon tissues. The fourth column on the right shows the FPKM values of FAS RNA-seq data. The box plot on top of each heatmaps summarize the statistical difference between normal and tumor samples for CpG site methylation and FAS mRNA expression. The p-values indicate the Welch’s t-test results. (**D**) Negative correlation between methylation of the three CpG sites as shown in B of the left three columns and FAS mRNA expression as shown in B of the right column.

Analysis of human cancer genomics database revealed that the FAS locus is not focally amplified across a dataset of 3131 tumors, suggesting that FAS is unlikely an oncogene. The FAS locus is significantly focally deleted across the entire dataset of these same 3131 tumors, including human colorectal cancer (Broad Institute tumorscape database, https://portals.broadinstitute.org/tcga/home; accessed on 25 November 2021), suggesting that FAS is likely a colorectal tumor suppressor in humans. Analysis of FAS expression level in different stages of human colorectal tumors indicated that FAS expression decreases with tumor progression, and the lowest FAS expression is in stage IV colorectal tumors (Figure 1A). Previous study has shown that FAS expression level in tumors with patient survival time indicates that a low FAS expression level is positively correlated with colorectal-cancer patient survival [42]. These findings indicate that FAS functions as a suppressor of human colorectal cancer.

### 3.2. FAS Expression Is Repressed by Its Promoter DNA Hypermethylation in Tumor Cells of Human Colorectal Cancer Patients

Literature indicates that FAS can be repressed by its promoter DNA hypermethylation in human colon tumor cell lines [36]. Analysis of the human genomic DNA sequence identified a CpG island surrounding the FAS transcription start site (Figure 1B). To determine the FAS promoter DNA methylation status in human colorectal cancer patients, DNA methylation datasets were extracted from TCGA database and analyzed for FAS promoter DNA methylation. DNA methylation peaks were detected in three CpG sites including the CpG island at the FAS promoter (Figure 1C). The FAS promoter DNA is hypermethylated in these three CpG sites in human colorectal tumors as compared to normal colon tissue (Figure 1C). Correlation of FAS mRNA expression level with the FAS promoter DNA hypermethylation level revealed that the FAS promoter DNA hypermethylation is inversely correlated with FAS expression level in tumors in human colorectal cancer patients (Figure 1D). Our findings indicate that FAS expression is repressed by its promoter DNA hypermethylation in human colorectal cancer patients.

### 3.3. Single-Cell RNA Sequencing Indicates That FAS Is Highly Expressed in Immune Cells and Normal Epithelial Cells in Human Colorectal-Cancer Patients

**Figure 2:**
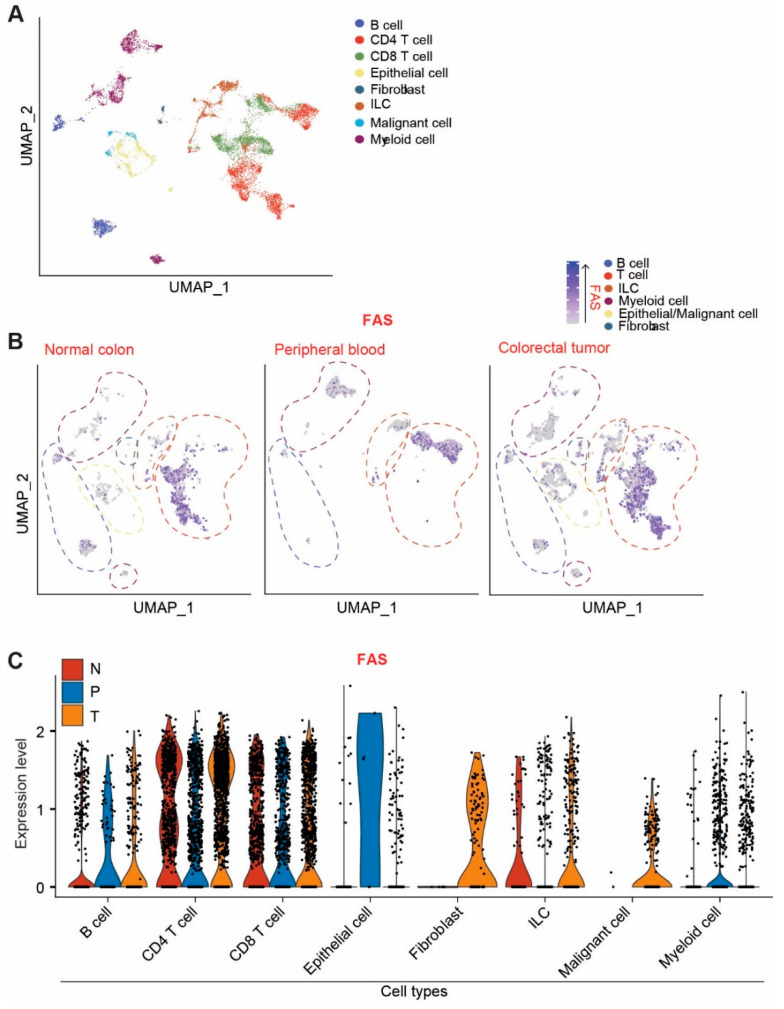
FAS expression profiles at the single-cell level in human colorectal-cancer patients. (**A**) UMAP projection of human colorectal-cancer scRNA-seq data. Original datasets were extracted from GSE146771 dataset. Cells are annotated according to dataset designation. (**B**) UMAP projection of FAS expression. Cells were subsetted by tissue of origin (normal colon, peripheral blood, and colorectal tumor). (**C**) FAS expression level in the indicated cell types in normal colon (N), peripheral blood (P), and colorectal tumor (T).

To determine the cellular FAS expression profiles in human colorectal-cancer patients, single-cell RNA-seq datasets were extracted from the raw datasets in the GEO database (GSE146771) [41] and analyzed for FAS expression at the single-cell level. Cell types that express FAS were annotated (Figure 2A). Normal colon tissues share similar FAS expression cellular patterns as colorectal tumor cells (Figure 2B). FAS expression was detected in all subsets of lymphocytes and epithelial cells. The FAS expression level is lower in colorectal tumor cells (Figure 2C).

### 3.4. Development of Cationic Lipid Encapsulated Codon-Optimized FAS cDNA-Expressing Plasmid DNA Nanoparticle

**Figure 3:**
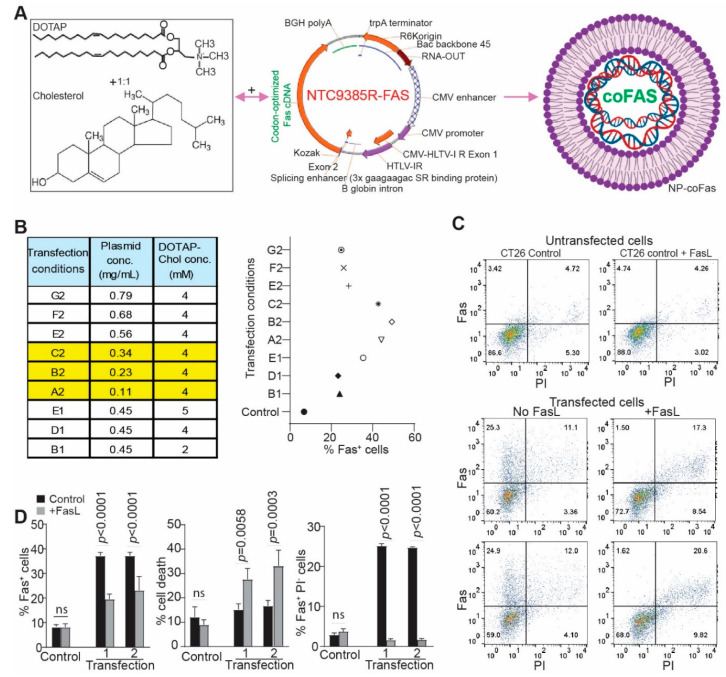
Restoring FAS expression enabled FASL-mediated elimination of FAS^+^ mouse colon-tumor cells in vitro. (**A**) DOTAP and cholesterol (1:1) cationic lipid (left panel) was used to encapsulate a codon-usage-optimized mouse FAS cDNA-expressing plasmid (middle panel) to produce the nanoparticle DOTAP-mFAS. (**B**) DOTAP-Cholesterol and the plasmid were formulated in different ratios as indicated (left table). The formulated DNA nanoparticles were then used to transfect mouse colon-tumor cells for 24 h. The transfected cells were analyzed for FAS expression on tumor-cell surface by flow cytometry. The FAS^+^ cells were quantified and presented on the right panel. (**C**) Representative dot plots of flow-cytometry data showing FAS expression in control and DOTAP-mFAS-transfected cells as described in (**B**). The number (1 and 2) under transfection indicates duplicated experiments. Shown are one of three independent experiments. (**D**) The transfected cells as shown in C were cultured with FASL for 24 h, stained with anti-FAS mAb and PI, and analyzed by flow cytometry. The indicated cell populations were quantified. The *p* value was determined by Student’s *t* test.

Cationic lipid-based nanoparticles are an efficient nucleic acid delivery system that selectively deliver DNA to tumors in vivo [43,44,45,46]. To restore FAS expression, we encapsulated FAS cDNA-expressing plasmids. Because FASL is primarily expressed on T cells, we first used an immune competent syngeneic colon-tumor mouse model. A codon-usage optimization strategy was undertaken to design and synthesize mouse FAS cDNA with optimized codon usage sequence to maximize protein expression (Figure S1). The codon-usage-optimized DNA were cloned into a nanoplasmid NTC9385R (Figure 3A). The plasmid DNA was then encapsulated by cationic lipids (DOTAP-Chol, 1:1) to formulate nanoparticles (DOTAP-Chol-mFAS) (Figure 3A).

### 3.5. Restoring FAS Expression Overcomes Colon Tumor Resistance to FASL-Induced Apoptosis In Vitro

The mouse colon tumor CT26 cell line has an MSS genotype and is highly metastatic [47,48,49]. CT26 cells express lower levels of FAS than the MSI subtype of MC38 tumor cells (Figure S2A,B) and are resistant to FASL-induced apoptosis (Figure S2C,D) [42]. We therefore sought to restore FAS expression in CT26 cells using codon-usage-optimized mFAS. Titration of DNA and DOTAP-Chol formulation achieved as high as 50% FAS^+^ cells (Figure 3B,C). Exogenous restoration of FAS in CT26 cells rendered tumor cell sensitivity to FASL-induced apoptosis (Figure 3C). All FAS^+^ tumor cells were effectively eliminated by FASL in vitro (Figure 3C,D). Taken together, we determined that restoring FAS expression in mouse colon-tumor cells is sufficient to render FASL-resistant metastatic mouse colon-tumor cells sensitive to FASL-induced apoptosis in vitro.

### 3.6. FAS DNA Nanoparticle Therapy Is Sufficient to Suppress Mouse Colon-Tumor Growth in Immune-Competent Mice

**Figure 4:**
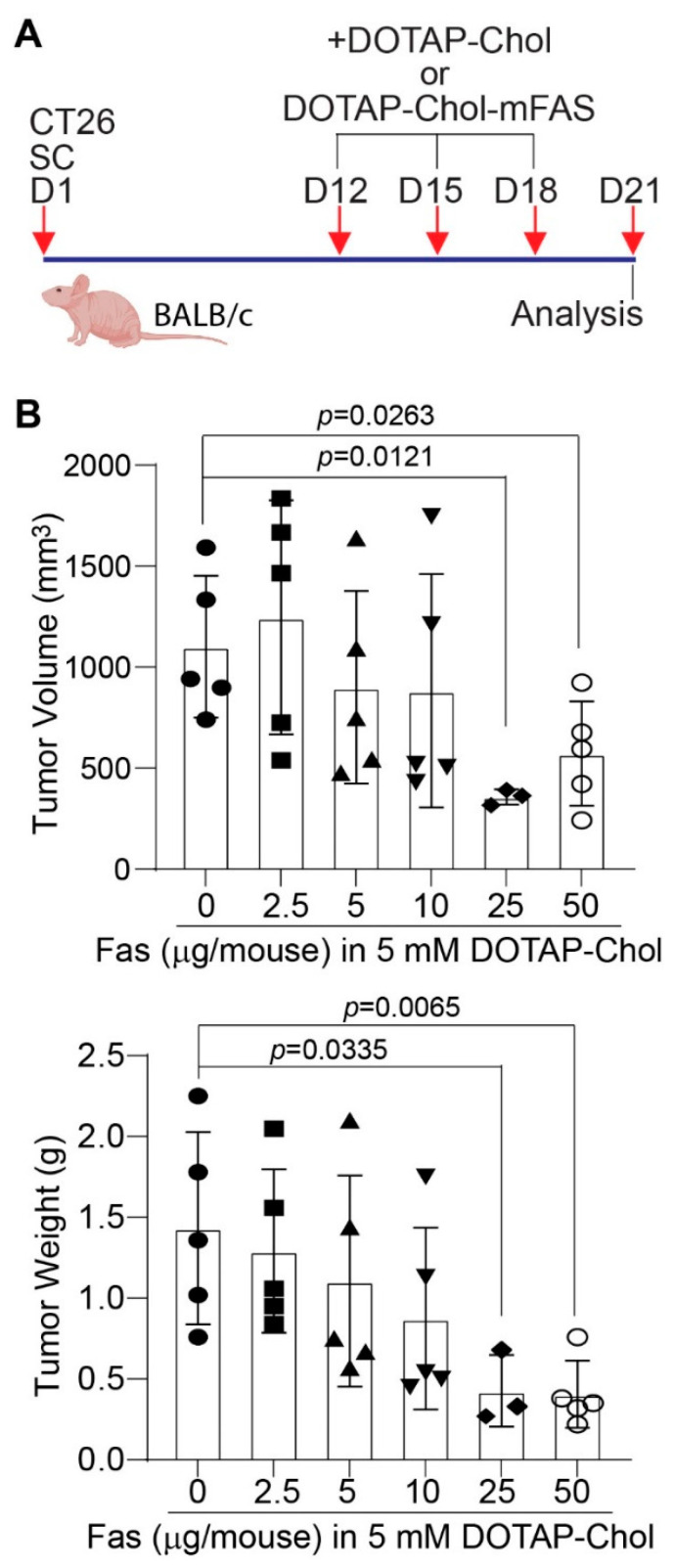
Dose response of mouse colon tumor to DOTAP-Chol-mFAS in tumor-bearing mice. (**A**) CT26 cells were injected to mice subcutaneously. Shown is the study design. (**B**) Tumor-bearing mice were randomized into six groups and treated with DOTAP-Chol-mFAS nanoparticle with various codon-usage-optimized mouse FAS cDNA-expressing plasmid doses as indicated. Tumor size and weight were analyzed at the end of the experiments.

To determine whether the above finding can be translated to colon-tumor growth suppression in vivo, we treated CT26 tumor-bearing mice with various doses of DOTAP-Chol-mFAS (Figure 4A). A mFAS dose-dependent suppression of tumor growth was observed, and a dose of 25 µg mFAS DNA encapsulated in 4 mM DOTAP-Chol achieved the highest tumor suppression efficacy (Figure 4B).

**Figure 5:**
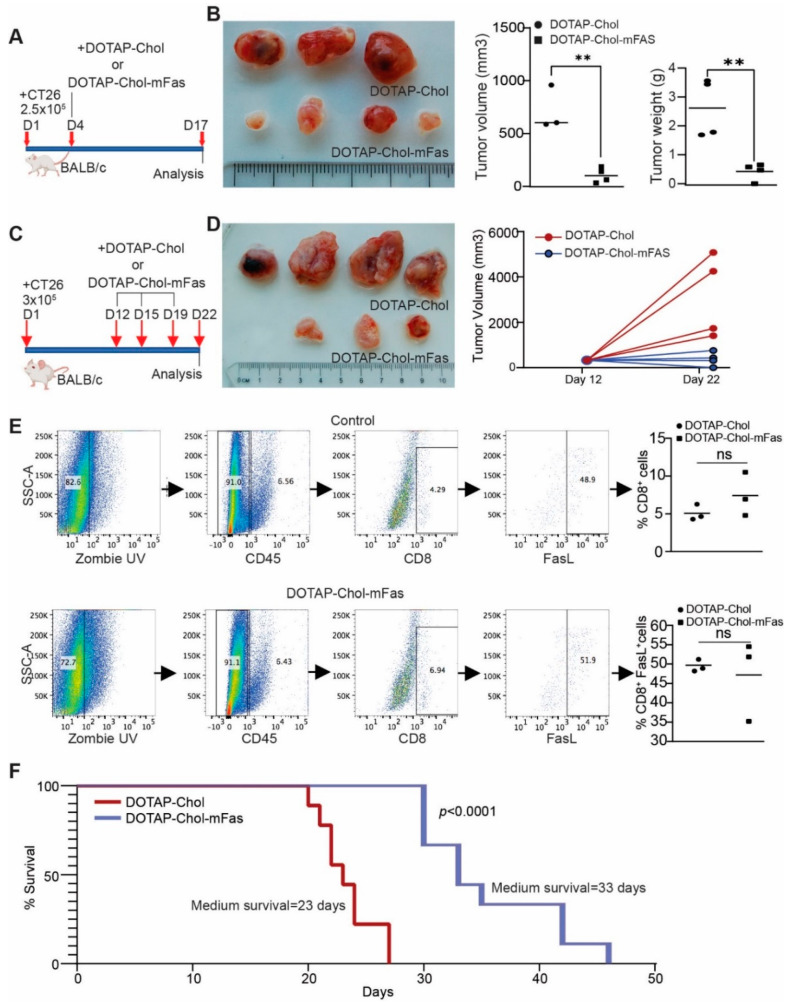
DOTAP-mFAS gene immunotherapy suppresses colon tumor growth in immune competent mice. (**A**) CT26 tumor cells were injected into mice subcutaneously. The mice were treated with DOTAP-mFAS nanoparticles starting at day 4 and treated as indicated. (**B**) Tumors were analyzed for size and weight. (**C**) CT26 tumor cells were injected into mice as in (**A**), and mice were treated as indicated. (**D**) Tumor growth was analyzed at the start of treatment and at the end of the experiment. (**E**) Tumors as shown in D were collected and analyzed for CD8^+^ and FASL^+^ T cells as shown. (**F**) CT26 tumor cells were injected intravenously into mice. The tumor-bearing mouse cages were randomized and treated with DOTAP-Chol or DOTAP-mFAS starting at day 5 every 3 days for 3 times. The mice were then monitored for survival.

In human clinical trials, DOTAP-Chol was used as a control [43]. To be consistent with this clinical practice for future clinical use, DOTAP-Chol was used as control in this study. We next treated tumor-bearing mice at the early stage (Figure 5A). One dose of DOTAP-Chol-mFAS treatment significantly suppressed tumor growth (Figure 5B). We then allowed the tumor to grow to a larger size before the treatment and treated the tumor-bearing mice three times with DOTAP-Chol-mFAS (Figure 5C). DOTAP-Chol-mFAS also significantly suppressed growth of the established large tumors (Figure 5D). Tumor tissues were then collected and analyzed for CD8^+^ T cell-tumor infiltration and FASL expression. As expected, no significant change in tumor-infiltration CTL level was observed, and approximately 50% of the colon tumor-infiltrating CTLs are FASL^+^ (Figure 5E). To further determine the function of FAS in colon-tumor suppression, we used an experimental lung-metastasis mouse model by injecting CT26 cells into mouse-tail veins. The tumor-bearing mice were treated with DOTAP-Chol-mFAS every three days three times. Mice in the control group had a median survival of 23 days. Mice in the treatment group had a median survival of 33 days (Figure 5F). Taken together, we determined that DOTAP-Chol-mFAS therapy is sufficient to suppress colon-tumor growth in vivo.

### 3.7. Exogenous Expression of Codon-Usage-Optimized FAS Induces Metastatic Human Colon-Tumor-Cell FAS Receptor Auto-Oligomerization and Tumor-Cell Auto-Apoptosis In Vitro

**Figure 6:**
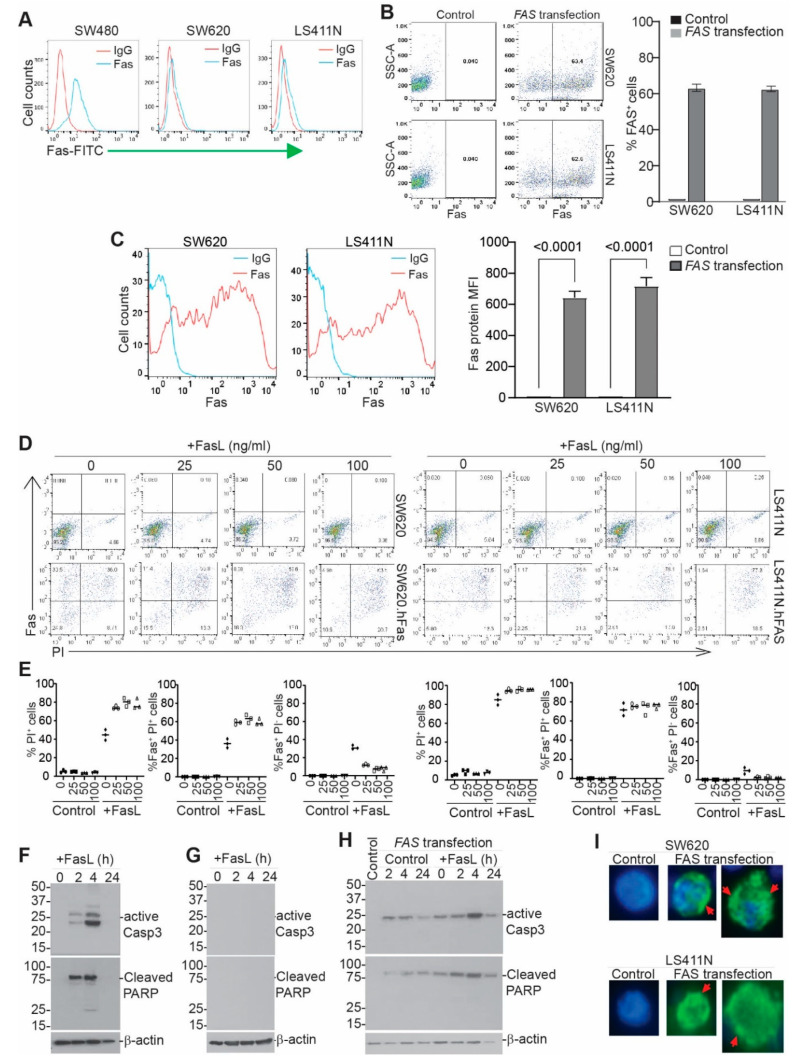
Restoring FAS expression induces colon-tumor-cell auto-apoptosis. (**A**) FAS expression in human colon-tumor cells. (**B**) Tumor cells were transfected with codon-optimized FAS cDNA-expressing plasmid. Shown are FAS protein dot plots and quantification of %FAS^+^ cells. Shown is one of three experiments. (**C**) The FAS^+^ cells as shown in B were gated and quantified for FAS protein mean fluorescent intensity (MFI). The left panel shows histography; the right panel shows FAS protein MFI. (**D**) Tumor cells were transfected as in B, cultured in the presence of FASL for 24 h, stained with FAS antibody and PI, and analyzed by flow cytometry. Shown are representative dot plots. (**E**) The indicated cell populations were quantified. (**F**,**G**) SW480 (F) and SW620 (**G**) cells were cultured in the presence of FASL (100 ng/mL); collected at 0, 2, 4, and 24 h; and analyzed by Western blotting. The blot was probed sequentially with anti-cleaved caspase 3, cleaved PARP, and β-actin. (**H**) SW620 cells were transfected with hFAS and analyzed by Western blotting as in F. (**I**) Control cells and hFAS-transfected SW620 and LS411N cells were analyzed by immunofluorescence for FAS protein (Green). Red arrows point to FAS aggregations. Blue is nucleus staining.

SW480 and SW620 cell lines are a matched pair of human primary and metastatic colon-tumor cell lines established from the same patient [50]. FAS expression is lower in SW620 cells than in SW480 cells (Figure 6A). Human colon-tumor cell line LS411N is a high-grade primary colon-tumor cell line that also has lost FAS expression (Figure 6A). Both SW620 and LS411N are resistant to FASL-induced apoptosis (Figure S3). To restore FAS expression in human colon-tumor cells, we synthesized human FAS cDNA with optimized codon use to maximize FAS protein expression. The synthesized gene was cloned to construct a DNA plasmid using a bacterial expression system. The plasmid DNA was then used to transfect SW620 and LS411N cells, resulting in FAS expression in over 60% of tumor cells (Figure 6B) and a high level of FAS protein on the tumor cell surface (Figure 6C). Analysis of tumor-cell death revealed that the transfected cells die in the absence of FASL, and cell death is further increased by FASL in vitro (Figure 6D,E). Almost all FAS^+^ cells are apoptotic (Figure 6D,E). FASL-induced rapid caspase 3 activation and PARP cleavage in SW480 cells (Figure 6F) but not in SW620 cells (Figure 6G). Exogenous expression of the codon-usage-optimized FAS cDNA resulted in caspase 3 activation and PARP cleavage in the absence of exogenous FASL (Figure 6H). A clear caspase 3 cleavage kinetics was observed, and caspase 3 was completed and cleaved 24 h later likely due to cell death (Figure 6F). One possible explanation for the above phenomenon is that tumor-cell-produced FASL engages FAS to kill these tumor cells. To test this hypothesis, we added human FASL-specific monoclonal antibody to the cell culture. Repeat attempts with this FASL blockade approach failed to block tumor-cell apoptosis of the tumor cells that express codon-usage-optimized FAS (Figure S4). We therefore termed this phenomenon “auto-apoptosis.” FASL enhances this process (Figure 6G). FASL binding to FAS receptor induces FASL monomer trimerization, followed by trimer oligomerization to initiate the death signal [51]. Analysis of FAS protein distribution indicates that exogenous expression of codon-optimized FAS results in FAS oligomerization in the absence of FASL (Figure 6I and Figure S5), a phenomenon we termed “auto-oligomerization.”

### 3.8. Restoring FAS Expression Is Sufficient to Suppress Metastatic Human Colon-Tumor Xenograft Growth In Vivo

To determine whether the above finding can be translated to human colon-tumor growth suppression in vivo, we produced a cationic lipid-encapsulated codon-usage-optimized human FAS cDNA nanoparticle (DOTAP-Chol-hFAS) (Figure S6). SW620 cells were injected into athymic nude mice to established tumor xenografts. To determine whether mouse FASL is capable of inducing human tumor-cell FAS-mediated apoptosis, the FASL-sensitive human Jurkat cells and mouse EL4 cells were used (Figure S7A,B). Human LS411N cells and mouse CT26 cells have no cell surface FAS protein (Figure S7C) but have abundant cellular FASL protein (Figure S7D). Co-culturing Jurkat cells with LS411N resulted in Jurkat cell apoptosis and co-culturing EL4 cells with CT26 cells resulted in EL4 cells apoptosis (Figure S7E). Mouse MC38 cells induced Jurkat cell apoptosis when co-cultured together (Figure S8A). Blocking FASL with a mouse FASL neutralization monoclonal antibody decreased Jurkat cell apoptosis (Figure S8B,C), albeit at a lower level.

**Figure 7:**
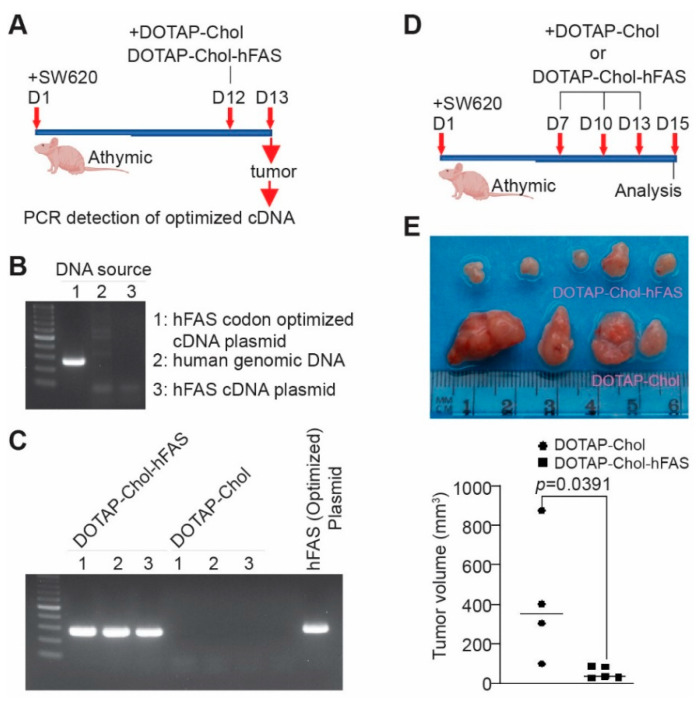
DOTAP-hFAS gene therapy suppresses human metastatic colon-tumor xenograft growth in athymic mice. (**A**) SW620 cells were injected subcutaneously to athymic nude mice. The tumor-bearing mice were treated as indicated. Tumors were collected for genomic DNA isolation. (**B**) Codon-optimized FAS cDNA plasmid, human tumor-cell genomic DNA, and human FAS cDNA plasmid were used as templates for PCR analysis using primers that are specific for the codon-optimized FAS cDNA. (**C**) Genomic DNA from xenografts as shown in A was analyzed by PCR using primers that are specific for the codon-optimized FAS cDNA. The hFAS codon-optimized cDNA plasmid was used as a positive control. (**D**) SW620 cells were injected into athymic nude mice to establish xenografts. The tumor-bearing mice were treated as shown. (**E**) The top panel shows the tumor xenografts. The lower panel represents the quantified tumor volume.

To determine the delivery efficiency of hFAS cDNA to the tumor site, we treated the xenograft-bearing mice with DOTAP-Chol-hFAS nanoparticles, collected tumor tissues one day later, isolated genomic DNA, and performed PCR using a pair of PCR primers that are specific for the codon-optimized human FAS cDNA sequence (Figure 7A). This PCR specifically detects the codon-sequence-optimized hFAS cDNA, not the endogenous FAS gene (Figure 7B). High levels of exogenous FAS were detected in the xenografts from mice treated with DOTAP-Chol-hFAS (Figure 7C), indicating efficient delivery of the codon-usage-optimized FAS cDNA to the tumor site. We then established human colon tumor xenografts and treated the tumor-bearing mice with DOTAP-Chol-hFAS as shown (Figure 7D). DOTAP-Chol-hFAS therapy is sufficient to suppress the xenograft tumor growth in vivo (Figure 7E). 

### 3.9. FAS DNA Nanoparticle Therapy Has No Significant Toxicity

FASL therapy causes lethal liver toxicity [52]. We therefore performed a preliminary toxicity study to determine whether DOTAP-Chol-FAS gene therapy has liver toxicity. Mice were treated with DOTAP-Chol-mFAS every three days for a total of three doses. Serum was collected for liver enzyme profiles to determine if liver-tissue damage was observed. No significant increases in liver enzymes were observed in the serum (Table S1). Liver tissues were analyzed by histological means and examined by a board-certified pathologist. No microscopic tissue injury was observed in the treated mice (Figure S9). We therefore conclude that DOTAP-Chol-mFAS therapy is safe to the liver.

## 4. Discussion

The FAS-mediated apoptosis-pathway activation initiates from FASL binding to the death receptor FAS, resulting in sequential signaling cascades including FAS receptor trimerization, oligomerization, death-inducing signaling-complex (DISC) formation, caspase activation, and cell death [51]. This signaling pathway is regulated by both pro- and anti-apoptotic protein factors [53,54]. In cancer cells, anti-apoptotic factors are often up-regulated and confer tumor-cell resistance not only to death-receptor-mediated apoptosis but also to death induction by chemotherapy, radiotherapy, and immunotherapy [7,13,14,55,56]. In human colorectal cancer, literature and our data indicate that FAS acts a tumor suppressor [7,15,16,17,18,19,20,21,22,42,54,57,58,59], and tumor cells may use silencing of FAS expression as a mechanism to advance the disease [20]. Consistent with the role of FAS in tumor cell apoptosis, emerging experimental data determined that FAS and other death pathways are essential for CAR-T cell efficacy [10,11,60,61]. A recent CRISPR screen identified an essential role for FAS-FASL in antigen-specific T cell killing, and tumor-cell eradication by tumor-specific CTLs and CAR-T cells was abrogated in FAS-deficient target cells despite the presence of activated and functional CTLs in vitro and in vivo [11]. Tumoral FAS expression alone is sufficient to predict the survival of CAR-T-treated human cancer patients [11]. Moreover, FAS-dependent tumor killing by CAR-T is augmented by inhibiting or knocking out anti-apoptotic regulators or by increasing pro-apoptotic regulators of the FAS signaling pathway [11,60,61]. An essential question then is whether restoring FAS expression alone is sufficient to suppress colon tumor growth. We determined in this study that restoring FAS expression alone is sufficient to suppress established metastatic colon tumor xenograft growth in vivo. 

Codon-usage optimization resulted in high levels of FAS protein on the metastatic colon tumor cell surface after ectopic expression. Strikingly, high levels of FAS expression lead to FAS receptor oligomerization in the absence of exogenous FASL in vitro, a phenomenon that we termed auto-oligomerization. Furthermore, this auto-oligomerization is followed by caspase 3 activation, PARP cleavage, and tumor cell death in the absence of exogenous FASL, a phenomenon we termed auto-apoptosis. It is known that tumor cells, including colon-tumor cells, express and release soluble FASL [62]. Consistent with this phenomenon, we observed high intracellular FASL protein in colon-tumor cells. However, neutralization of FASL failed to decrease tumor cell death, indicating that tumor-cell-produced FASL plays no significant role in tumor-cell FAS-mediated apoptosis, and the auto-oligomerization and auto-apoptosis are not caused by tumor-cell-produced FASL. It is therefore reasonable to conclude that ectopic expression of codon-usage-optimized FAS results in high levels of FAS receptor on the metastatic tumor cell surface, which undergoes receptor auto-oligomerization and resultant auto-apoptosis in vitro. This auto-apoptosis might be responsible for the efficacy of DOTAP-Chol-hFAS therapy in the immune-deficient athymic mice. The molecular mechanism underlying this auto-oligomerization and auto-apoptosis remains to be determined.

Multiple epigenetic mechanisms are involved in the silencing of FAS in the tumor cells [20,37] (Figure 2). These epigenetic mechanisms may compensate for each other in the suppression of FAS expression, which may limit the efficacy of epigenetic agent monotherapy. The recent breakthrough of the SARS-CoV2 mRNA-based vaccine highlights the significance of nucleic-acid-based therapy. Lipid nanoparticles such as DOTAP-Cholesterol are an effective carrier of nucleic acid for selective delivery of nucleic acids into tumor cells [43,44,45,46]. Using this nanoparticle delivery system in combination with codon-usage-optimized FAS cDNA, we determined that tumor-selective lipid-nanoparticle-FAS-cDNA-targeted delivery is sufficient to restore FAS expression to suppress metastatic human colon tumor xenograft growth in vivo in the absence of immune cells and thereby in the absence of membrane-bound FASL. FASL is expressed on activated T cells and NK cells under physiopathological conditions. T cells and NK cells are often suppressed in the tumor microenvironment [63]. Our findings indicate that DOTAP-Chol-hFAS therapy is sufficient to suppress human colon tumor growth in the immune-suppressive tumor microenvironment. DOTAP-Chol-hFAS is therefore potentially an effective agent to restore FAS expression in metastatic human colorectal carcinoma to suppress metastasis.

## 5. Conclusions

FAS expression decreases with tumor progression in human colorectal cancer, and the FAS expression level is positively correlated with human colorectal-cancer patient survival time. FAS is silenced by its promoter DNA hypermethylation in human colorectal carcinoma. We determined that ectopic expression of FAS is sufficient to sensitize colon-tumor cells to apoptosis induction in vitro. Furthermore, lipid nanoparticle encapsulated FAS-encoding DNA plasmid effectively restored FAS expression in mouse colon-tumor cells in immune-competent mice and in human colon-tumor cells in a xenograft mouse model, respectively, resulting in tumor-growth suppression in vivo. Our data determine that tumor-selective delivery of FAS DNA nanoparticles is sufficient and yet safe for suppression of human colon-tumor growth in vivo.

## Data Availability

The datasets and materials used during the current study are available from the corresponding author on request.

## Supplementary Materials

The following are available online at https://www.mdpi.com/article/10.3390/cancers14020361/s1, Figures S1–S9: Figure S1. Codon usage optimized mouse FAS-coding sequence. Figure S2. FAS expression and tumor cell sensitivity to FAS-induced apoptosis in vitro. Figure S3. Differential sensitivity of human colon tumor cell lines to FASL-induced apoptosis. Figure S4. Human colon tumor cell-produced FASL has no effect on human tumor FAS-mediated apoptosis. Figure S5. Overexpression of FAS results in FAS protein aggregation on tumor cell surface. Figure S6. Codon usage optimized human FAS-coding sequence. Figure S7. Tumor cells express FASL and kill FAS-expressing tumor cells. Figure S8. Mouse tumor cell expressed FASL induces FAS-mediated apoptosis in human tumor cells. Figure S9. Liver toxicity analysis of cationic lipid-encapsulated FAS DNA nanoparticle therapy. Figure S10. Western blots. A & B. Figure S11. Agarose gel images of FAS PCR. Table S1. Mouse liver enzyme profiles.
