# Supplementary material for: Restoring FAS Expression via Lipid-Encapsulated FAS DNA Nanoparticle Delivery Is Sufficient to Suppress Colon Tumor Growth In Vivo
Source: Cancers (Basel). 2022 Jan 12;14(2):361. doi: 10.3390/cancers14020361 (PMC8773494; doi:10.3390/cancers14020361)
Supplement: Supplementary file 1 [file cancers-14-00361-s001.zip › cancers-1502489-supplementary.pdf]

```
        10         20         30         40         50         60         70         80
ACGCGTGTCG ACGCCACCAT GCTTTGGATT TGGGCTGTGC TGCCTCTGGT GCTGGCTGGA TCTCAACTGA GAGTGCACAC
                       M  L  W  I   W  A  V  L  P  L  V   L  A  G   S  Q  L   R  V  H T

        90        100        110        120        130        140        150        160
CCAGGGCACC AACAGCATCA GCGAGAGCCT GAAGCTGCGG AGAAGAGTGC GGGAAACCGA CAAGAATTGC AGCGAGGGCC
 Q  G  T   N  S  I   S  E  S  L   K  L  R   R  R  V   R  E  T  D   K  N  C   S  E  G

       170        180        190        200        210        220        230        240
TGTACCAAGG CGGCCCATTT TGCTGTCAGC CTTGCCAGCC AGGCAAGAAG AAGGTCGAGG ACTGCAAGAT GAACGGCGGC
 L  Y  Q  G   G  P  F   C  C  Q   P  C  Q  P   G  K  K   K  V  E   D  C  K  M   N  G G

       250        260        270        280        290        300        310        320
ACCCCTACAT GTGCCCCTTG TACAGAGGGC AAAGAGTACA TGGACAAGAA CCACTACGCC GACAAGTGCA GACGGTGCAC
 T  P  T   C  A  P  C   T  E  G   K  E  Y   M  D  K  N   H  Y  A   D  K  C   R  R C T

       330        340        350        360        370        380        390        400
CCTGTGTGAT GAGGAACACG GCCTGGAAGT GGAAACAAAC TGCACCCTGA CACAGAACAC CAAGTGCAAG TGCAAACCCG
 L  C  D   E  E  H   G  L  E  V   E  T  N   C  T  L   T  Q  N  T   K  C  K   C  K  P

       410        420        430        440        450        460        470        480
ACTTCTACTG CGACAGCCCT GGCTGCGAGC ACTGTGTCAG ATGTGCCTCT TGCGAGCACG GCACCCTGGA ACCATGTACC
 D  F  Y  C   D  S  P   G  C  E   H  C  V  R   C  A  S   C  E  H   G  T  L  E   P C T

       490        500        510        520        530        540        550        560
GCCACCAGCA ACACCAACTG CAGAAAGCAG AGCCCCAGAA ACCGGCTGTG GCTGCTGACA ATCCTGGTGC TGCTGATCCC
 A  T  S   N  T  N  C   R  K  Q   S  P  R   N  R  L  W   L  L  T   I  L  V   L  L  I  P>

       570        580        590        600        610        620        630        640
TCTGGTGTTC ATCTACCGGA AGTACAGAAA GCGGAAGTGC TGGAAGCGGA GACAGGACGA CCCTGAGAGC AGAACCAGCA
  L  V  F   I  Y  R   K  Y  R  K   R  K  C   W  K  R   R  Q  D  D   P  E  S   R  T  S

       650        660        670        680        690        700        710        720
GCAGAGAAAC AATCCCCATG AACGCCAGCA ACCTGAGCCT GAGCAAGTAC ATCCCCAGAA TCGCCGAGGA CATGACCATC
S  R  E  T   I  P  M   N  A  S   N  L  S  L   S  K  Y   I  P  R   I  A  E  D   M  T  I

       730        740        750        760        770        780        790        800
CAAGAGGCCA AGAAGTTCGC CCGCGAGAAC AACATCAAAG AGGGCAAGAT CGACGAGATC ATGCACGACA GCATCCAGGAQ
    E  A   K  K  F  A   R  E  N   N  I  K   E  G  K  I   D  E  I   M  H  D   S  I  Q  D

       810        820        830        840        850        860        870        880
CACCGCCGAG CAGAAAGTTC AGCTGCTGCT GTGCTGGTAT CAGAGCCACG GCAAGTCCGA CGCCTACCAG GATCTGATCA
 T  A  E   Q  K  V   Q  L  L  L   C  W  Y   Q  S  H   G  K  S  D   A  Y  Q   D  L  I

       890        900        910        920        930        940        950        960
AGGGCCTGAA GAAAGCCGAG TGCAGAAGAA CCCTGGACAA GTTCCAGGAC ATGGTGCAGA AGGATCTGGG CAAGAGCACC
K  G  L  K   K  A  E   C  R  R   T  L  D  K   F  Q  D   M  V  Q   K  D  L  G   K  S T

       970        980        990       1000       1010
CCTGACACCG GCAATGAGAA TGAGGGCCAG TGCCTGGAAT GATGAGAATT CAGATCT
 P  D  T   G  N  E  N   E  G  Q   C  L  E   *  *
```

1-6 MluI [ACGCGT]
7-12 SalI [GTCGAC]
1006-1011 EcoR1 [GAATTC]
1012-1016 BglII [AGATCT]

Figure S1. **Codon usage optimized mouse Fas-coding sequence**. The yellow fonts indicate cloning sites. The top line is the codon usage optimized sequence. The bottom is the Fas protein sequence.

Supplemental Data

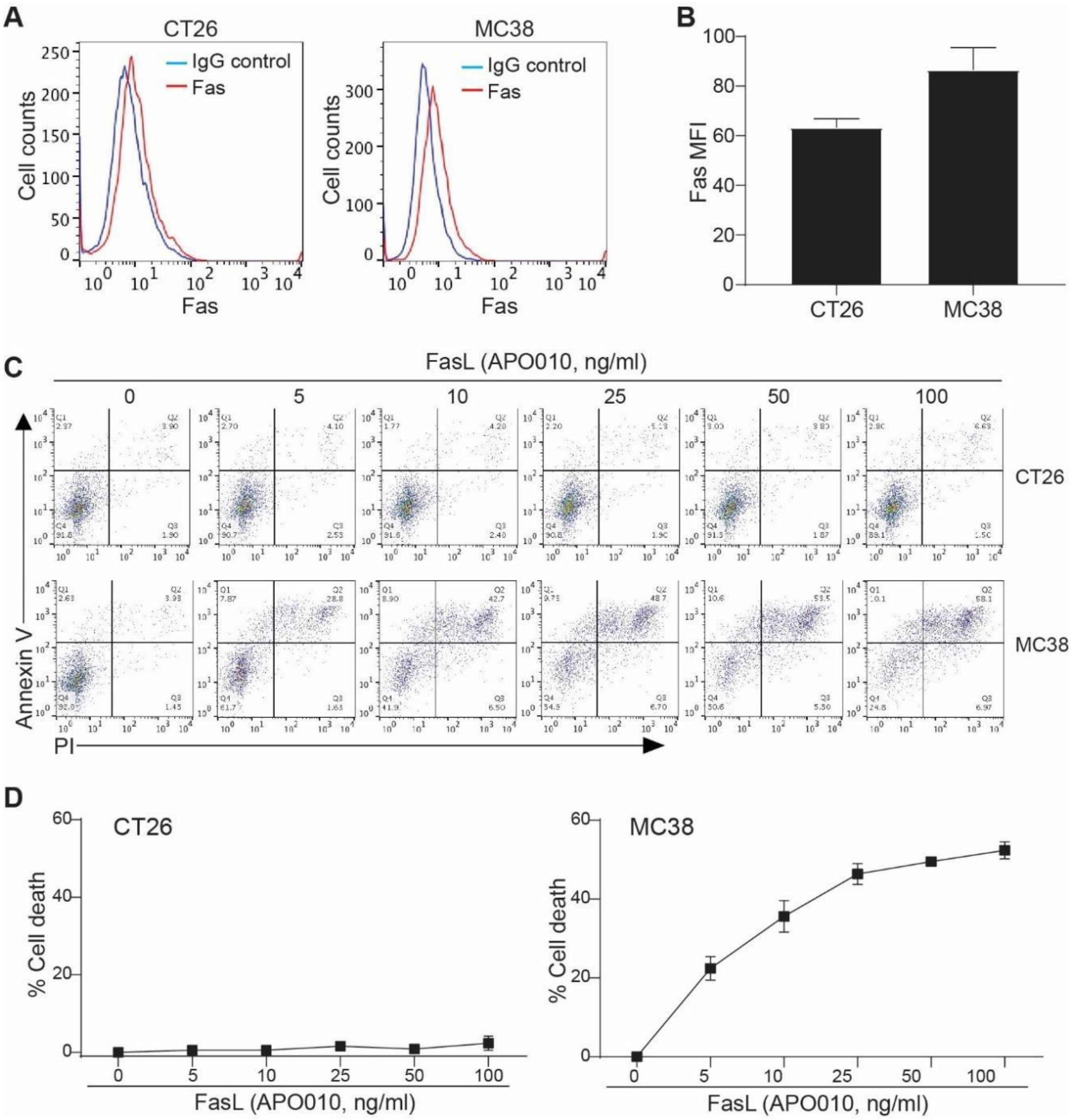

Figure S2. **Fas expression and tumor cell sensitivity to FasL-induced apoptosis in vitro**. **A**. Mouse colon tumor cell lines CT26 and MC38 were stained with IgG isotype control or Fas-specific antibody, and analyzed by flow cytometry. Shown are representative histographs of Fas protein staining. **B**. Fas MFI as shown in A is quantified. CT26 and MC38 cells were cultured in the presence of FasL at the indicated concentrations for 24h. Cells were then stained with Annexin V and PI, and analyzed by flow cytometry. D. Cell death as shown in C is quantified as % Annexin V^{+} PI^{+} cells. The untreated cells are set as background.

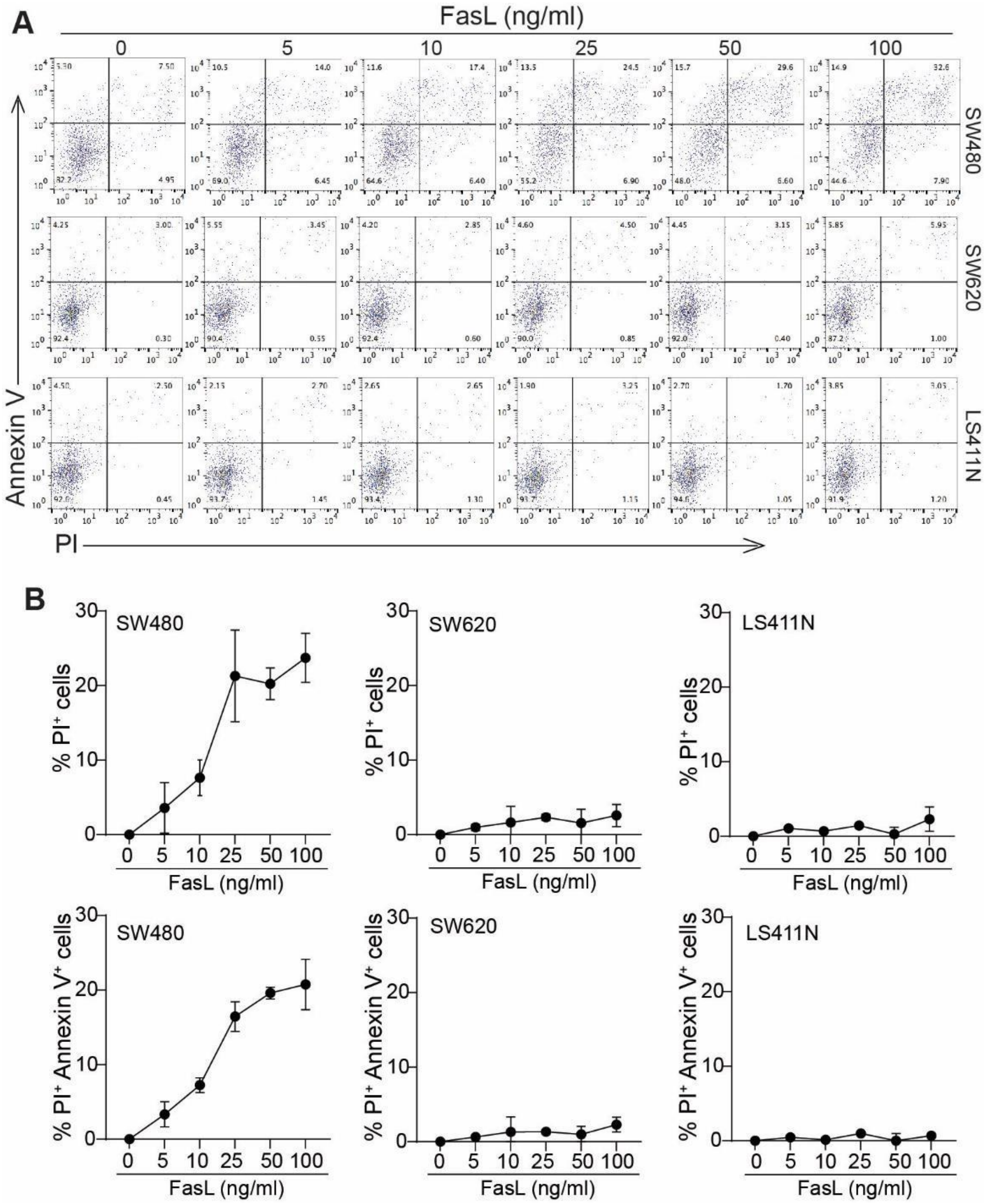

Figure S3. **Differential sensitivity of human colon tumor cell lines to FasL-induced apoptosis**. **A**. Tumor cells were cultured in the presence of FasL for 24h. Cells were collected, stained with Annexin V and PI, and analyzed by flow cytometry. Shown are representative dot plots. **B**. Cell cells were quantified.

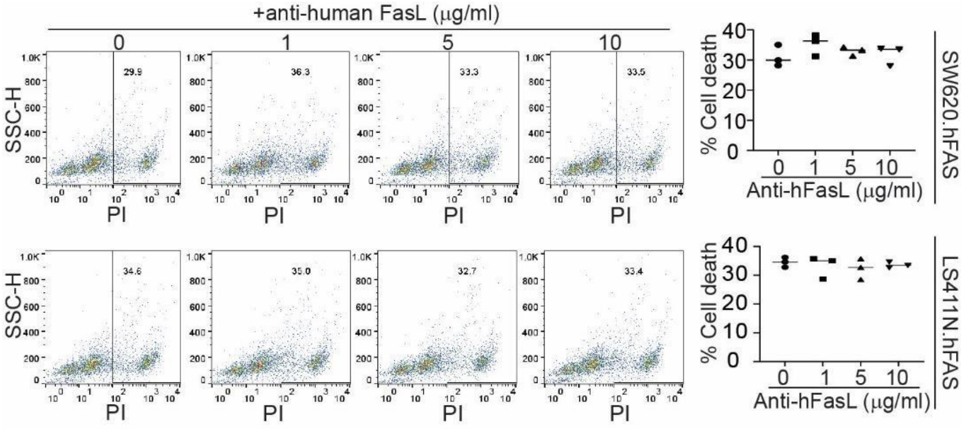

Figure S4. Human colon tumor cell-produced FasL has no effect of human tumor cell Fas-mediated apoptosis. SW620 and LS411N cells were transfected with the codon usage-optimized human *FAS* cDNA-expressing plasmid overnight. The transfected cells were cultured in the presence of human FasL neutralization monoclonal antibody at the indicated concentrations for 24h. Cells were collected, stained with Annexin V and PI, and analyzed by flow cytometry. Shown are representative dot plots (left panel) and quantification of % cell death (right panel).

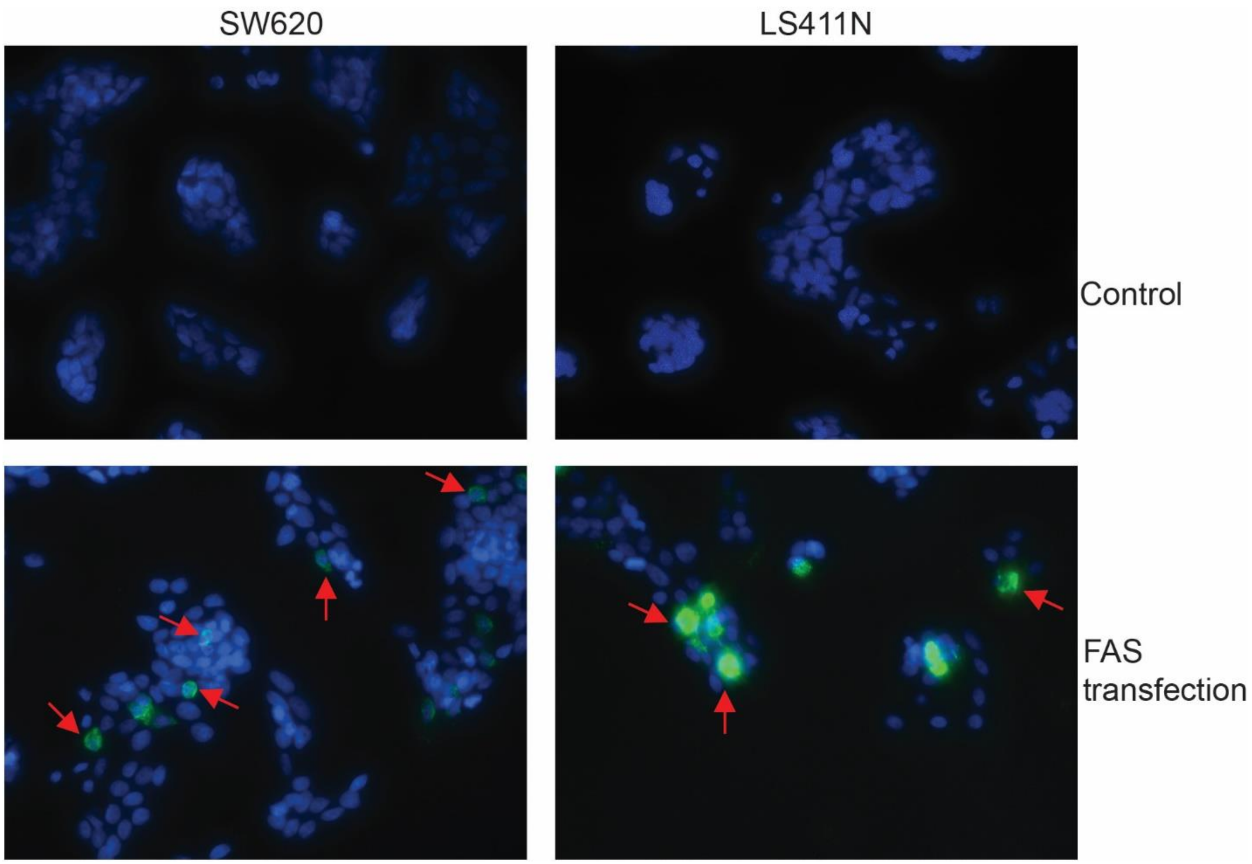

Figure S5. Overexpression of Fas results in Fas protein aggregation on tumor cell surface. SW620 and LS411N cells were transfected with the codon usage optimized human Fas cDNA-encoding plasmid. Control cells and hFAS-transfected SW620 and LS411N cells were analyzed by immunofluorescence for FAS protein (Green). Blue is nucleus staining. Red arrows point to FAS aggregations. Shown are overlay. This figure is related to Figure 6I.

ORF	Protected sites
19-1029 [ATG...TGA]	1-6 MluI [ACGCGT]<br>7-12 SalI [GTCGAC]<br>1030-1035 EcoRI [GAATTC]<br>1036-1041 BglII [AGATCT]

```
                                    M  L  G  I     T  L  L  P  L  V  L  T  S  V  A  R
   1.  ACGCGTGTCGACGCCACCATGCTCGGAATTTGGACACTGCTGCCTCTGGTGCTGACCAGCGTGGCAAGA
       L  S  S  K  S  V  N  A  Q  V  T  D  I  N  S  K  G  L  E  L  R  K  T
  70.  CTGAGCAGCAAGAGCGTGAACGCCCAAGTGACCGACATCAACAGCAAAGGCCTGGAACTGAGAAAGACC
       V  T  T  V  E  T  Q  N  L  E  G  L  H  H  D  G  Q  F  C  H  K  P  C
 139.  GTGACCACCGTGGAAACCCAGAACCTGGAAGGCCTGCACCACGACGGCCAGTTCTGTCACAAACCTTGT
       P  P  G  E  R  K  A  R  D  C  T  V  N  G  D  E  P  D  C  V  P  C  Q
 208.  CCACCTGGCGAGCGGAAGGCCAGAGATTGCACAGTGAATGGCGACGAGCCTGACTGCGTGCCCTGTCAA
       E  G  K  E  Y  T  D  K  A  H  F  S  S  K  C  R  R  C  R  L  C  D  E
 277.  GAGGGCAAAGAGTACACCGACAAGGCCCACTTCAGCAGCAAGTGCAGACGGTGCAGACTGTGCGACGAA
       G  H  G  L  E  V  E  I  N  C  T  R  T  Q  N  T  K  C  R  C  K  P  N
 346.  GGCCACGGACTGGAAGTGGAAATCAACTGCACCCGGACACAGAACACCAAGTGCCGGTGCAAGCCCAAC
       F  F  C  N  S  T  V  C  E  H  C  D  P  C  T  K  C  E  H  G  I  I  K
 415.  TTCTTCTGCAACAGCACCGTGTGCGAGCACTGCGACCCTTGTACCAAGTGCGAACACGGCATCATCAAA
       E  C  T  L  T  S  N  T  K  C  K  E  E  G  S  R  S  N  L  G     L  C
 484.  GAGTGCACCCTGACCTCCAACACGAAGTGCAAAGAGGAAGGCAGCAGAAGCAACCTCGGCTGGCTGTGT
       L  L  L  L  P  I  P  L  I  V     V  K  R  K  E  V  Q  K  T  C  R  K
 553.  CTGCTGCTGCTCCCCATTCCTCTGATCGTGTGGGTCAAGCGGAAAGAGGTGCAGAAAACCTGCCGGAAG
       H  R  K  E  N  Q  G  S  H  E  S  P  T  L  N  P  E  T  V  A  I  N  L
 622.  CACCGGAAAGAGAACCAGGGCTCTCACGAGTCCCCAACACTGAACCCTGAGACAGTGGCCATCAACCTG
       S  D  V  D  L  S  K  Y  I  T  T  I  A  G  V  M  T  L  S  Q  V  K  G
 691.  AGCGACGTGGACCTGAGCAAGTACATCACCACAATCGCCGGCGTGATGACCCTGAGCCAAGTGAAGGGA
       F  V  R  K  N  G  V  N  E  A  K  I  D  E  I  K  N  D  N  V  Q  D  T
 760.  TTCGTGCGGAAGAACGGCGTGAACGAGGCCAAGATCGACGAGATCAAGAACGACAACGTGCAGGACACC
       A  E  Q  K  V  Q  L  L  R  N     H  Q  L  H  G  K  K  E  A  Y  D  T
 829.  GCCGAGCAGAAGGTTCAGCTGCTGAGAAACTGGCACCAGCTGCACGGCAAGAAAGAGGCCTACGACACC
       L  I  K  D  L  K  K  A  N  L  C  T  L  A  E  K  I  Q  T  I  I  L  K
 898.  CTGATCAAGGACCTGAAGAAAGCCAACCTGTGTACCCTGGCCGAGAAGATCCAGACCATCATCCTGAAG
       D  I  T  S  D  S  E  N  S  N  F  R  N  E  I  Q  S  L  V  *  *
 967.  GACATCACCAGCGACAGCGAGAACAGCAACTTCCGGAACGAGATCCAGAGCCTGGTGTAATGAGAATTC

1036.  AGATCT
```

Figure S6. Codon usage optimized human FAS-coding sequence. The yellow fonts indicate cloning sites. The protein sequence is under the nucleotide sequence.

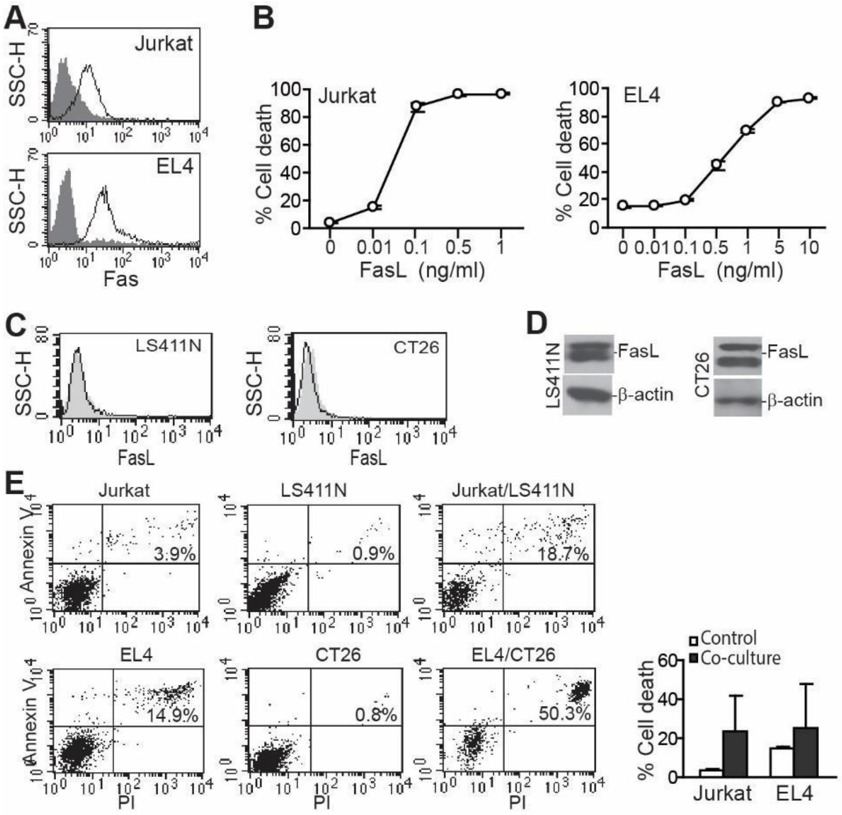

Figure S7. Tumor cells express FasL and kill Fas-expressing tumor cells. A. Jurkat and EL4 cellswere stained with IgG istype control antibody (gray area) or Fas-specific antibody (black line), and analyzed by flow cytometry. Shown are representative histographs of Fas protein staining. **B**. Jurkat and EL4 cells were cultured in the presence of FasL at the indicated concentrations for 24h. Cells were collected and stained with Annexin V and PI. The stained cells were then analyzed by flow cytometry. Shown are quantification of cell death. **C**. LS411N and CT26 cells were stained for Fas protein as in A. **D**. LS411N and CT26 cells were analyzed by Western blotting and sequentially probed with FasL-specific antibody and β-actin. **E**. Jurkat and LS411N cells were cultured either alone or co-cultured for 24h. EL4 and CT26 cells were also cultured either alone or co-cultured for 24h. The culture supernatants were collected, stained with Annexin V and PI, and analyzed by flow cytometry. Right panel: representative dot plots. % cells death (Annexin V^+ PI^+) was quantified are presented at the right.

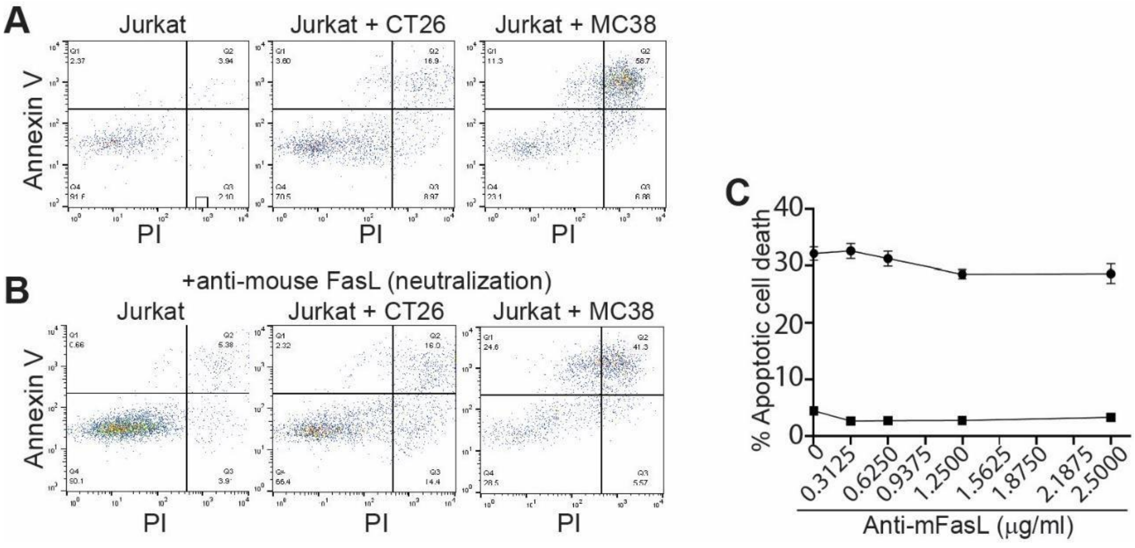

Figure S8. Mouse tumor cell expressed FasL induces Fas-mediated apoptosis in human tumor cells. A. Jukart cells were co-cultured with CT26 and MC38 cells, respectively, for 24h. Culture supernatant was collected, stained with Annexin V and PI, and analyzed by flow cytometry. **B**. Jurkat cells were co-cultured with CT26 and MC38 as in A in the presence of mouse FasL neutralization monoclonal antibody for 24h, and analyzed by flow cytometry as in A. Shown are representative dot plots. **C**. The co-culture supernatant of Jurkat and MC38 as shown in B were quantified. The bottom line is Jurkat cell death without co-cultured tumor cells. The top line is Jukat cell death in the presence of MC38 cell co-culture and mouse FasL neutralization antibody at the indicated concentrations.

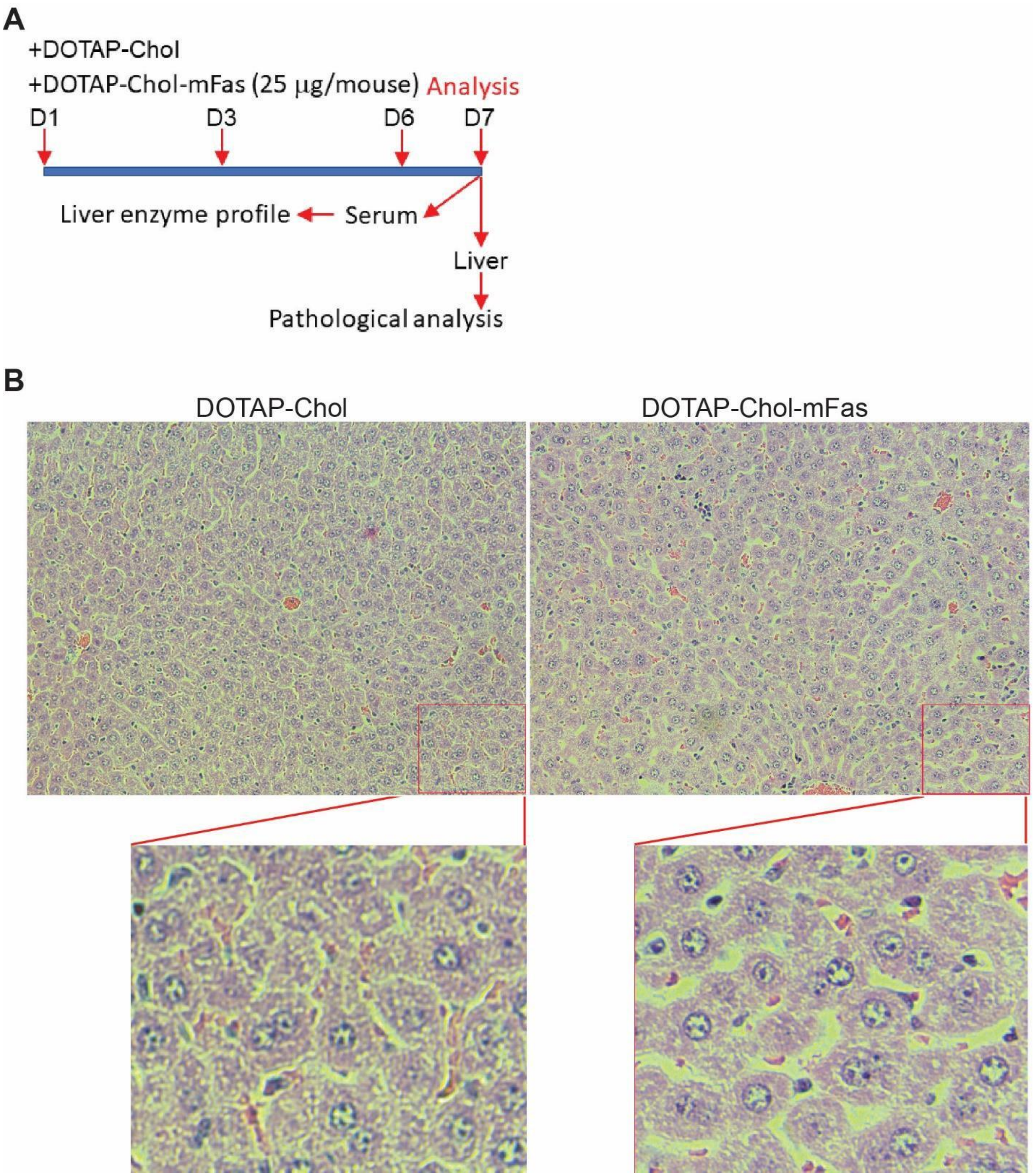

Figure S9. **Liver toxicity analysis of cationic lipid-encapsulated Fas DNA nanoparticle therapy.** Top panel: study design. Bottom panel: tumor-free BALB/c mice were treated with control nanoparticle (DOTAP-Chol) or cationic lipid-encapsulated *Fas* nanoparticle as shown in the top panel. Liver tissues were collected, fixed, embedded, sectioned, and stained with H&E. Shown are liver cells.

Figure S10

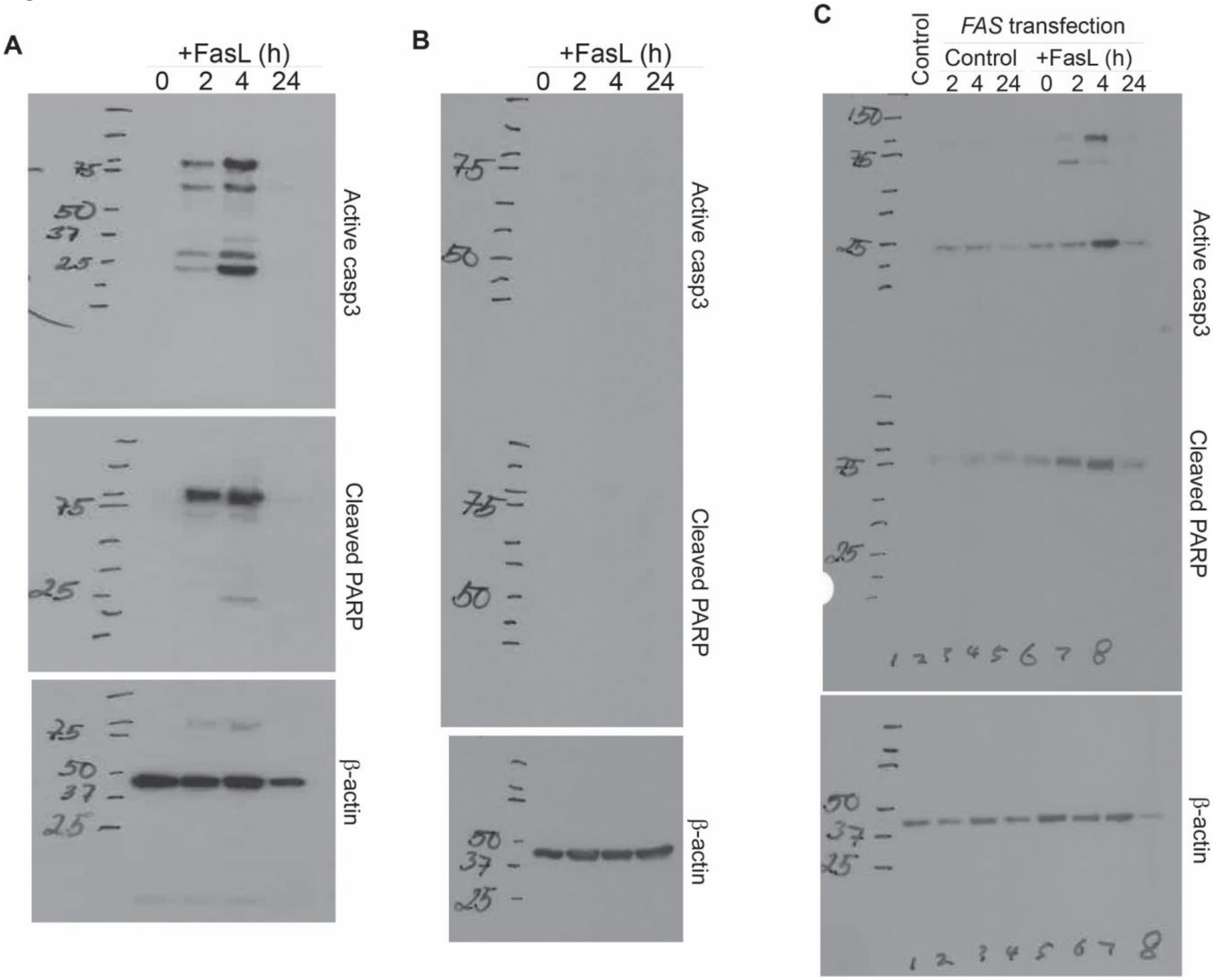

Figure S10. Western blots. A & B. SW480 (A) and SW620 (B) cells were cultured in the presence of FasL (100 ng/mL); collected at 0, 2, 4, and 24 h; and analyzed by Western blotting. The blot was probed sequentially with anti-cleaved caspase 3, cleaved PARP, and β-actin. **C**. SW620 cells were transfected with hFAS, treated as shown, and analyzed by Western blotting as in B. This figure is related to Figure 6F-H.

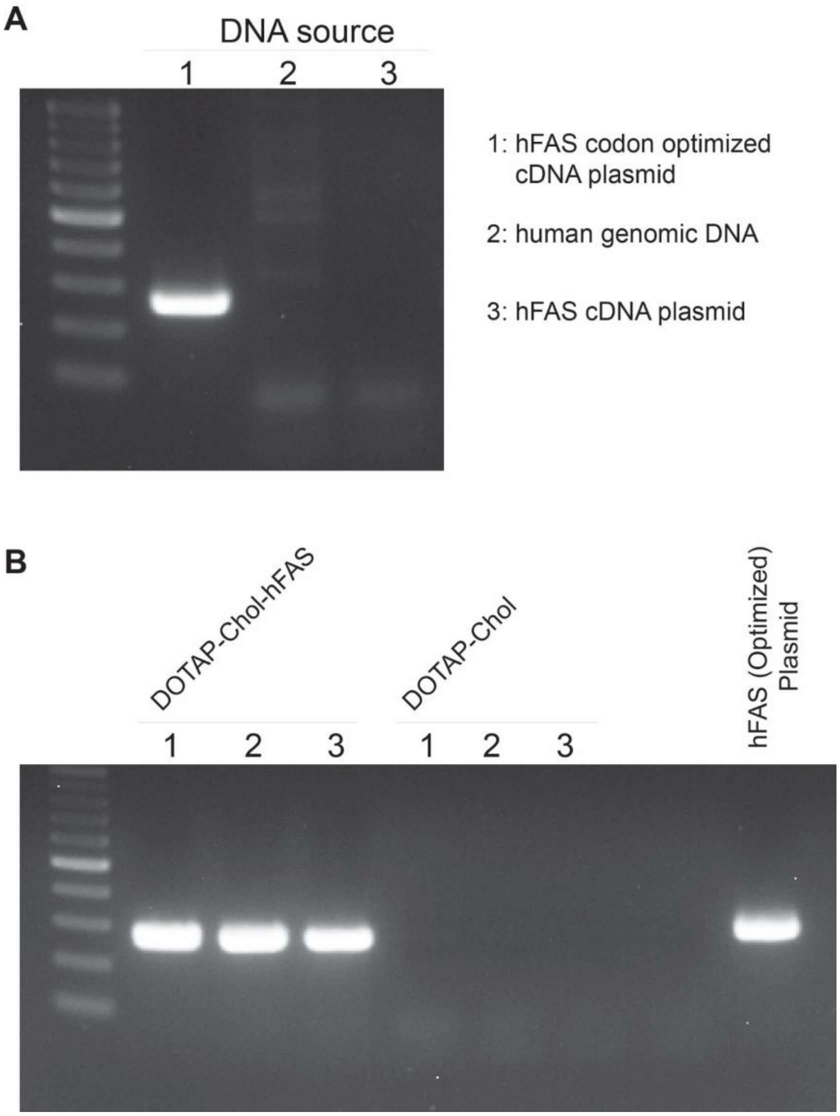

Figure S11. Agarose gel images of *FAS* PCR. **A**. Codon-optimized *FAS* cDNA plasmid, human tumor-cell genomic DNA, and human *FAS* cDNA plasmid were used as templates for PCR analysis using primers that are specific for the codon-optimized *FAS* cDNA. **B**. Genomic DNA from xenografts was analyzed by PCR using primers that are specific for the codon-optimized *FAS* cDNA. The hFAS codon-optimized cDNA plasmid was used as a positive control. This figure is related to Figure 7B & C.

Table S1. Mouse liver enzyme profile

Enzyme/Protein	DOTAP-Chol	mFAS-DOTAP-Chol	*p* valus
Total Protein g/dl	6.525 +/- 0.28	6.175 +/- 0.51	0.287059
Albumin g/dl	3.625 +/- 0.17	3.24+/- 0.33	0.062612
Alk Phos U/L	143 +/- 24.12	52.2 +/- 13.37	0.001697
ALT U/L	33 +/- 7.52	52.4 +/- 27.41	0.193929
AST U/L	261.26 +/- 112.35	257.75 +/- 153.82	0.971984
Cholesterol mg/dl	147.5 +/- 15.07	124.6 +/- 20.55	0.09545
Total Bilirubin mg/dl	0.25 +/- 0.6	0.325 +/- 0.15	0.405172
GGT U/L	5 +/- 1.83	9.33 +/- 7.57	0.42709
